# Prevalence, current status, and prevention of dental extractions in dogs: A retrospective study

**DOI:** 10.17221/40/2024-VETMED

**Published:** 2025-01-17

**Authors:** Kwangil Han, Zhenglin Piao, Chul Park, Md. Mahbubur Rahman, Namsoo Kim

**Affiliations:** ^1^Department of Veterinary Surgery, College of Veterinary Medicine, Chonbuk National University, Iksan City, Jeollabuk do, Republic of Korea; ^2^Department of Veterinary Dentistry, Royal Animal Medical Center, Jungnang-gu, Seoul, Republic of Korea; ^3^Department of Veterinary Medicine, College of Agriculture, Yanbian University, Yanji, P.R. China; ^4^Gachon Pain Center and Department of Physiology, College of Medicine, Gachon University, Incheon, Republic of Korea; Kwangil Han, Zhenglin Piao, Chul Park and Md. Mahbubur Rahman contributed equally to this work

**Keywords:** age, dental extraction, dogs, prevalence, preventive

## Abstract

Dental health has historically received little attention in veterinary medicine, but is becoming more common. This study aimed to report the prevalence of dental extractions in dogs in Seoul, Republic of Korea, describe the current status of dental health and determine any preventive methods. In total, 166 dogs participated in the study, presented to the veterinary hospital with an oral disorder or for a routine check-up were included in the study. Teeth were extracted from 130 dogs (78.32%). A single tooth was extracted from 18 dogs (13.85%), whereas multiple teeth (2–29 teeth) were extracted from 112 dogs (86.15%). Ten teeth were extracted in 31 dogs (27.67%). In descending order, the most extracted teeth were PM2, PM3, PM4, and PM1. The age at the first dental check-up, the average interval between dental check-ups, and the average interval between the previous two dental check-ups in the extraction group were significantly greater than those in the non-extraction group. In contrast, the number of dental check-ups was significantly lower. In conclusion, this study suggests regular dental check-ups to prevent dental extractions. The data provide useful information for veterinary dental health management and the prevention of tooth extractions.

Dental diseases are generally managed with medicinal treatment of periodontitis, endodontic treatment, and tooth extraction (Bellows et al. 2019). Dental extraction or exodontia is a surgical process in which one or more teeth are removed from their sockets. Teeth extraction in animals is usually recommended when the supporting structures, such as the cementum, periodontal ligament, and alveolar bone, are severely damaged and cannot be stabilised by specialised care (Gengler 2013). There are numerous indications for dental surgeons to perform exodontia, such as irreparable tooth damage, periodontal disease, decayed teeth, dental injuries, excessive tooth movement, dental abscesses, orthodontic disorders, impacted teeth, and occasional tumours.

The periodontium is a rich and extensive network of blood vessels through which the tooth mobility in its socket allows bacteria and their by-products to enter the lymphatic and circulatory systems. As a result, the body’s immune response to these microorganisms can form immune complexes in the bloodstream (Whyte et al. 2014). These complexes can internally attach to the walls of the endothelium, leading to malfunctions in the kidneys, liver, joints, and heart (Whyte et al. 2014; Pereira Dos Santos et al. 2019).

Therefore, dental and oral illnesses have been associated with systemic disorders such as hepatic or cardiovascular diseases (thromboembolic cardiac diseases, cerebral and myocardial infarctions, stroke, and atherosclerosis), diabetes mellitus, persistent obstructive pulmonary disease, endocarditis, and bacteraemia (Pereira Dos Santos et al. 2019; Sakai et al. 2019). Oral and dental diseases have been identified as a “silent epidemic” in the general population (Crall 2009) because the state of one’s oral health significantly impacts one’s overall health.

Dental health is less focused on in the veterinary profession despite its significant impact on the comfort and well-being of pets. Detecting oral diseases in dogs is challenging because a radiographic examination is required under general anaesthesia (Bellows et al. 2019). Pet owners often disagree with this procedure and base health assessments are taken on naked-eye visual examinations. Moreover, pets often hide their discomfort (Gengler 2013). Pets brought to veterinary hospitals with oral disorders tend to present with severe conditions, and veterinarians commonly advise tooth extractions after an oral radiographic evaluation. However, tooth extraction can affect the feeding behaviour and disrupt the balance of the stomatognathic system, leading to negative consequences, including gripping, mastication and showing self-power to other dogs. Therefore, it is important to minimise tooth extractions.

This study aimed to report the prevalence of dental extractions in dogs in Seoul, Republic of Korea, analyse the current state of dental health, and identify preventive measures for tooth extractions. Examining the relationships among various factors, this study aimed to raise awareness about tooth extractions among pet owners and veterinarians.

## MATERIAL AND METHODS

The clinical data were collected from a clinical database. The data were classified based on the sex, age, breed, number and location of the extracted teeth, frequency, and age at dental check-ups. This study used statistical methods to identify the correlations between tooth extraction and the above variables.

### Animals

The data were recorded during an 8-year period from January 2015 to December 2023, during which 166 dogs with oral disorders or for a routine check-up were brought to the Royal Animal Medical Center (RAMC), an animal hospital in Seoul, Republic of Korea. The Ethics Committee of the RAMC approved this investigation under Protocol No. 05/2024. Only the animals included in this study were subjected to a radiographic examination.

### Classification of dogs

The dogs were classified according to sex: male and female (Table 1) and according to the animal’s age (as reported by the owners): > 2 y, 2 to > 6 y, 6 to > 10 y, 10 to 16 y (Table 2) according to the breed (Table 3) to find out predisposing factors.

**Table 1:** Sex-dependent prevalence of the tooth extractions in dogs

Sex	Male	Female
Number	81	49
Percentage	62.31	37.69
Average age	7.68 ± 0.50	7.90 ± 0.60
Number of teeth extracted	5.63 ± 0.86	4.59 ± 1.08
Age at the first dental check-up	3.14 ± 0.31	3.03 ± 0.47
Number of dental check-ups	2.81 ± 0.26	3.29 ± 0.37
Average interval of the dental check-ups	3.54 ± 0.32	3.13 ± 0.33
Average interval of the last two dental check-ups	4.20 ± 0.38	4.02 ± 0.59

**Table 2:** Effects of the age and dental check-up status on the teeth extraction

Group	A (< 2 y)	B (2 to 6 y)	C (6 to 10 y)	D (10 to 16 y)
Number	13	38	33	48
Percentage	9.85	28.79	25.00	36.36***
Average age	1.00 ± 0.00	3.91 ± 0.15	7.48 ± 0.20	12.58 ± 0.25
Number of teeth extracted	2.77 ± 0.53	3.46 ± 1.01*	3.94 ± 0.84*	8.40 ± 1.36***
Age at the first dental check-up	1.00 ± 0.10	3.18 ± 0.20	3.08 ± 0.65	3.66 ± 0.51
Number of dental check-ups	1.00 ± 0.00	1.43 ± 0.12	3.31 ± 0.27	4.46 ± 0.44
Average interval of the dental check-ups	1.00 ± 0.00	3.15 ± 0.20	2.92 ± 0.30	4.53 ± 0.53**
Average interval of the last two dental check-ups	1.00 ± 0.00	2.92 ± 0.23*	3.74 ± 0.67**	6.23 ± 0.61***

Data are reported as means ± standard error of the mean. *P < 0.05, **P < 0.01, ***P < 0.001. Tukey’s multiple comparisons tests by one-way analysis of variance versus the A group

**Table 3:** Breed-dependent prevalence of the tooth extraction in the dogs

Breed	No. of animals	Percentage	Average age	No. of teeth extracted
Poodle	32	24.62	7.45 ± 0.61	5.28 ± 1.76
Maltese	13	10.00	8.85 ± 1.37	6.50 ± 1.86
Mixed	12	9.23	8.92 ± 1.62	7.25 ± 3.12
Yorkshire	9	6.92	7.00 ± 1.51	6.22 ± 1.69
Chihuahua	9	6.92	8.22 ± 1.66	7.11 ± 1.37
Shih Tzu	8	6.15	11.13 ± 1.27	5.00 ± 1.71
Schnauzer	6	4.62	12.13 ± 0.54	5.50 ± 2.09
Pekinese	6	4.62	6.83 ± 1.47	3.00 ± 2.24
Cocker Spaniel	5	3.85	10.80 ± 1.16	10.80 ± 7.49
Shiba Inu	5	3.85	2.00 ± 0.63	1.60 ± 0.24
Pomeranian	5	3.85	5.40 ± 1.33	3.75 ± 1.28
Bichon	5	3.85	4.80 ± 1.66	0.40 ± 0.24
Maltipoo	3	2.31	3.00 ± 1.00	3.33 ± 1.86
Spitz	2	1.54	7.5 ± 3.50	1.00 ± 0.00
Goldendoodle	1	0.77	1	1
Pompitz	1	0.77	1	1
Dachshund	1	0.77	14	6
Border Collie	1	0.77	6	1
Rottweiler	1	0.77	7	1
Welsh Corgi	1	0.77	6	1
Coton de Tulear	1	0.77	9	9
Miniature Pinscher	1	0.77	12	4

### Diagnosis

The clinical history and physical examination of the clinical signs and symptoms served as the basis for the primary diagnosis. The clinical history included a previous oral examination to remove the dental calculus and tooth brushing and identify any symptoms of oral abnormalities (bad breath, pain, or bleeding on one side). A confirmatory diagnosis was made using comprehensive diagnostic imaging reports after oral disinfection with anaesthesia, which included general photographs and radiographs.

**Figure 1:**
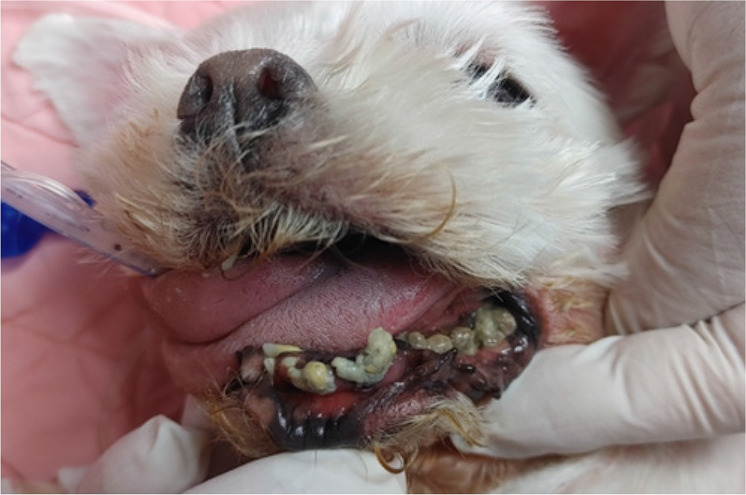
Tooth examination of a dog showing severe abnormalities

**Figure 2:**
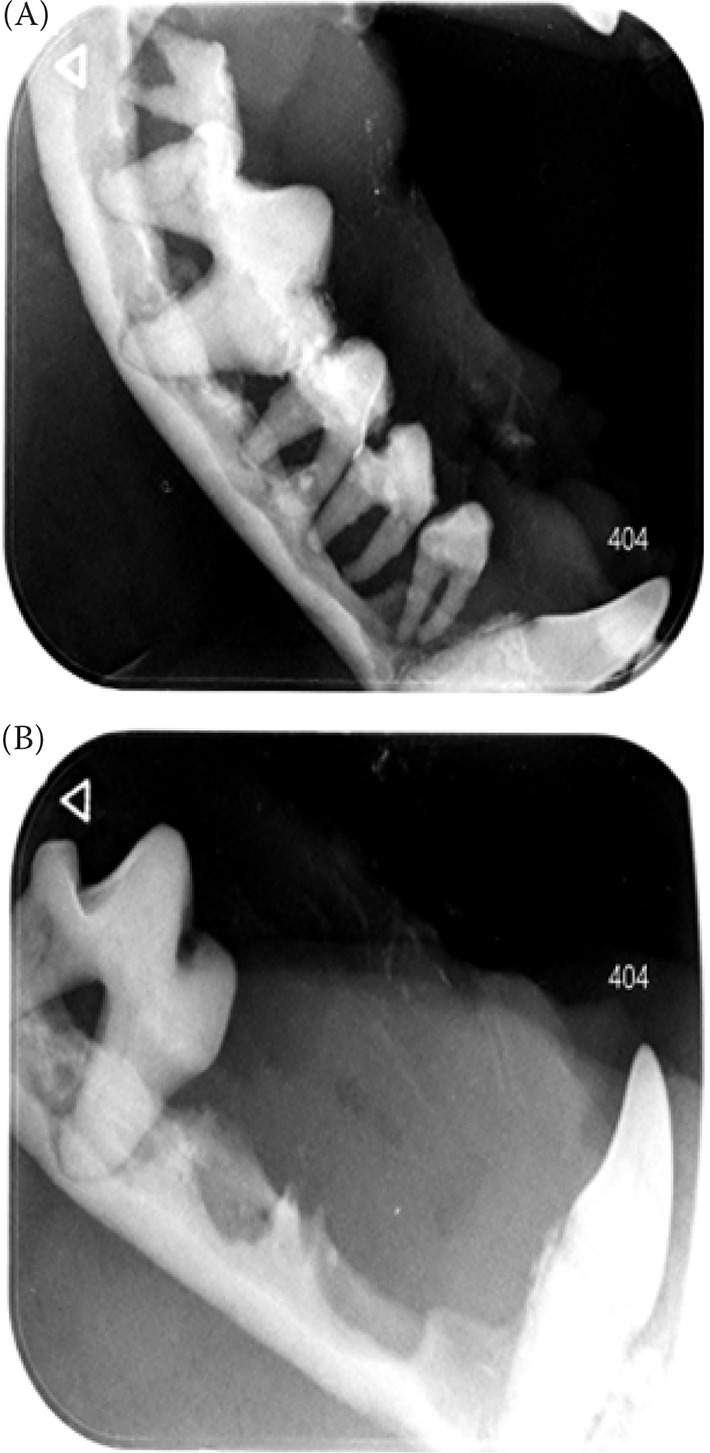
Preoperative (A) and postoperative (B) radiographs showing that the cementum, periodontal ligament, and alveolar bone were severely damaged

General photographs were obtained before and after the dental calculus removal, and dental radiographs were collected in the sternal/dorsal recumbent position. Veterinarians recorded the number and position of abnormal teeth, the severity and location of the periodontal disease, the presence of dental calculus, tooth wear (attrition), dental caries, and any other abnormalities in the oral cavity, such as the presence of oronasal fistulas, skin fistulas, pus, or tooth loss (Figures 1 and 2).

### Treatment

Following the diagnosis of dental disease, the dogs were treated according to the condition by treatment for periodontitis, endodontics, and exodontia/teeth extraction. The surgical procedure was performed after obtaining informed consent from the owners.

**Figure 3:**
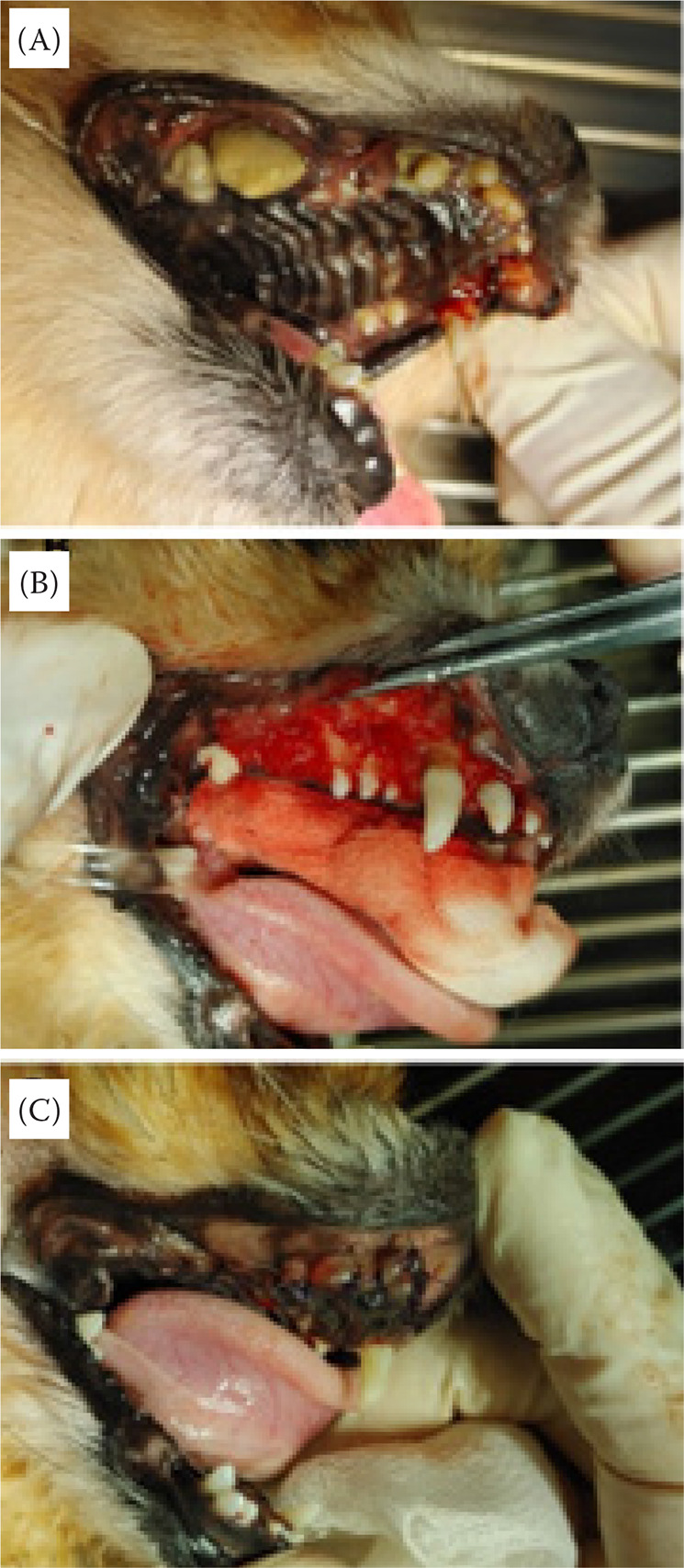
Preoperative (A), intraoperative (B) and postoperative (C) images of a tooth extraction of a dog

Anaesthesia was induced by administering propofol intravenously at a rate of 6–8 mg/kg, and sevoflurane (1–5%) was used to maintain the anaesthesia (Kim et al. 2020). A ventilation system maintained positive pressure, while automated anaesthetic equipment (PAIEON, J & TEC, Goyang-Si, Gyeonggi-do, Republic of Korea) monitored the electrocardiogram (ECG) and end-tidal CO_2_ levels during the operation (Jeong et al. 2019). Surgical procedures depend on the tooth’s location (Figure 3).

### Statistical analysis

The data were analysed using Prism v5.03 (Graph Pad Software Inc., San Diego, CA, USA) through a Bonferroni post hoc test after conducting a one-way analysis of variance (ANOVA) or Student’s *t*-test. The results were reported as the mean value plus or minus the standard error of the mean (SEM) or as a proportion. Correlations were assessed using Spearman’s rank correlation coefficients (Rahman et al. 2014). The level of significance was set at *P* < 0.05.

## RESULT

### Effect of the sex, age, and breed on the tooth extraction

In this study, their owners brought 166 dogs with oral disorders or for a routine check-up to a veterinary hospital. The result revealed that teeth were extracted from 130/166 dogs (78.32%), and 36/166 (21.69%) dogs were discharged following the radiographic examination and dental scaling. In the extraction group, 81 of 130 (62.31%) dogs were male and 49 (37.69%) were female (Table 1). Additionally, 124/130 (95.38%) patients underwent neutering. The age range was 1 to 18 years, averaging 7.76 ± 0.38 years. Group A (> 2 y) comprised 13/130 (9.85%), group B (2 to > 6 y) comprised 38/130 (28.79%), group C (6 to> 10 y) comprised 33/130 (25.00%), and group D (10 ≤ 16 y) comprised 48/130 (36.36%) dogs. The dog group most affected by the age was the 10 to 16 years old (Table 2). Interestingly, the number of teeth extractions increased with increased of age. The number of teeth extracted was significantly higher (*P* < 0.001) in dogs 10 to 16 y (8.40 ± 1.36) compared with those younger than 2 y (2.77 ± 0.53) (Table 2).

The breeds that were most frequently reported were Poodle 32/130 (24.62%), Maltese 13/130 (10.00%), mixed 12/130 (9.23%), Yorkshire 9/130 (6.92%), Chihuahua 9/130 (6.92%), Shih Tzu 8/130 (6.15%), Schnauzer 6/130 (4.62%), Pekinese 6/130 (4.62%), Cocker Spaniel 5/130 (3.85%), Shiba Inu 5/130 (3.85%), Pomeranian 5/130 (3.85%), Bichon 5/130 (3.85%), Maltipoo 3/130 (2.31%), Spitz 2/130 (1.54%), Goldendoodle 1/130 (0.77%), Pompitz 1/130 (0.77%), Dachshund 1/130 (0.77%), Border Collie 1/130 (0.77%), Rottweiler 1/130 (0.77%), Welsh Corgi 1/130 (0.77%), Coton de Tulear 1/130 (0.77%), and Miniature Pinscher 1/130 (0.77%) (Table 3).

### Number and sites of the tooth extraction

**Figure 4:**
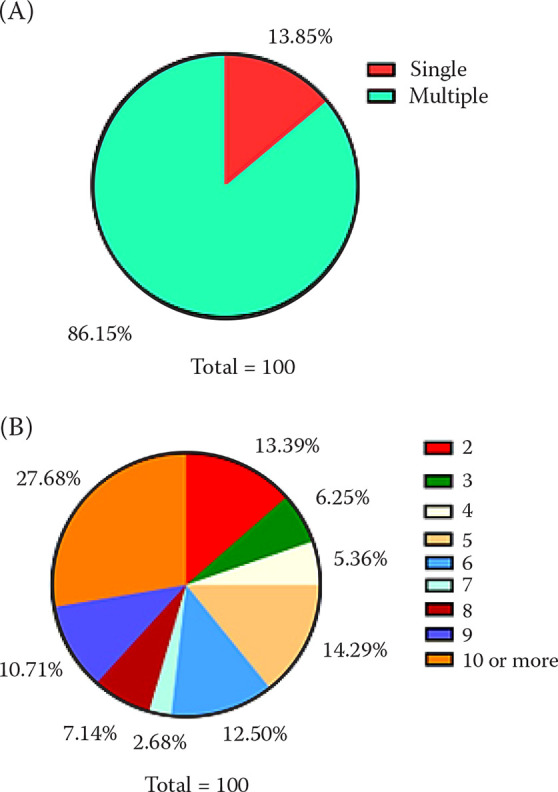
Prevalence and distribution of the number of teeth extracted by each case A single tooth was extracted from 13.85% of the dogs, while multiple teeth were extracted from 86.15% of the dogs (A). 2 teeth (13.39%), 3 teeth (6.25%), 4 teeth (5.36%), 5 teeth (12.50%), 6 teeth (2.68%), 7 teeth (7.14%), 8 teeth (7.14%), 9 teeth (10.71%) and 10 or more teeth were extracted from 27.68% of the dogs (B)

The number of teeth extracted from each dog ranged from 1–29, averaging 5.85 ± 0.54 teeth per dog. A single tooth was extracted from 18 dogs (13.85%), and multiple teeth were extracted from 112 dogs (86.15%). In the dogs with multiple teeth extractions; 2 teeth were extracted from 15/112 dogs (13.39%), 3 teeth were extracted from 7/112 dogs (6.25%), 4 teeth were extracted from 6/112 dogs (5.36%), 5 teeth were extracted from 16/112 dogs (14.29%), 6 teeth were extracted from 14/112 dogs (12.50%), 7 teeth were extracted from 3/112 dogs (2.68%), 8 teeth were extracted from 8/112 dogs (7.14%), 9 teeth were extracted from 12/112 dogs (10.71%), and 10 or more teeth were extracted from 31/112 dogs (27.68%) (Figures 3 and 4).

Regarding the total tooth extraction, the percentage of PM2 was 153/761 (20.11%), followed by PM3 at 146/761 (19.19%), PM4 at 92/761 (12.09%), and PM1 at 74/761 (9.72%). Of the most commonly extracted PM2 particles on the left side, 43 were in the maxilla and 46 were in the mandible; on the right side, these numbers were 30 and 34, respectively. In total, 373 (left = 199 and right = 174) teeth were extracted from the maxilla, and 388 (left = 212 and right = 176) were extracted from the mandible (Table 4).

**Table 4:** Number and percentage of the tooth extractions in the dogs according to the location

Maxilla	Right												Left										
		M2	M1	PM4	PM3	PM2	PM1	C	I3	I2	I1		I1	I2	I3	C	PM1	PM2	PM3	PM4	M1	M2	
No. of teeth extracted		15	13	20	31	30	20	15	12	8	10		8	10	9	13	18	43	41	23	18	16	
Percentage		11.54	9.85	15.15	23.48	22.73	15.15	11.36	9.09	6.06	7.58		6.06	7.58	6.82	9.85	13.64	32.58	31.06	17.42	13.64	12.12	
Teeth extracted in one site	174												199										
Teeth extracted in maxilla	373																						
Mandible	Right												Left										
	M3	M2	M1	PM4	PM3	PM2	PM1	C	I3	I2	I1		I1	I2	I3	C	PM1	PM2	PM3	PM4	M1	M2	M3
Total	7	16	11	26	31	34	17	11	7	8	8		10	10	8	13	19	46	43	23	17	18	5
Percentage	5.30	12.12	8.33	19.70	23.48	25.76	12.88	8.33	5.30	6.06	6.06		7.58	7.58	6.06	9.85	14.39	34.85	32.58	17.42	12.88	13.64	3.79
Teeth extracted in one site	176												212										
Teeth extracted in mandible	388																						
Total extracted teeth	761																						

### Factors influencing tooth deterioration

**Figure 5:**
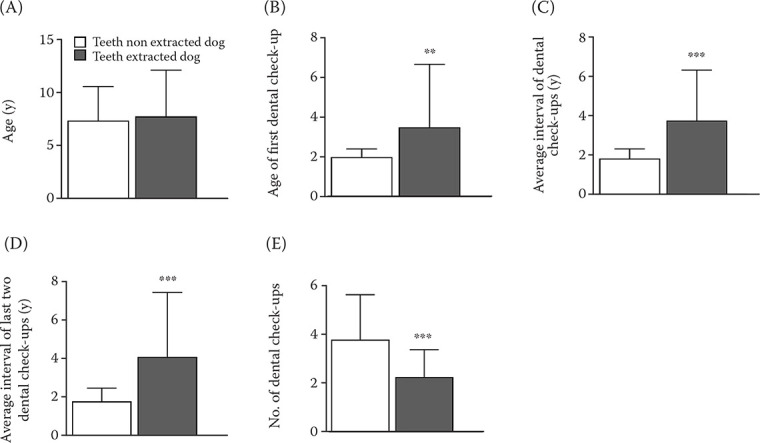
Factors influencing the tooth deterioration and extraction Age of the dogs (A), starting dental check-up time (B), the average interval of the dental check-up time in multiple check-ups (C), the average interval of the dental check-up time in the last two check-ups (D), number of dental check-ups (E)

There was no significant age difference between the extracted group (7.76 ± 0.38 y) and the non-extracted group (7.36 ± 0.53 y). However, the age at the first dental check-up (3.48 ± 0.28 y), average interval of the dental check-ups (3.76 ± 0.23 y), and average interval of the last two dental check-ups (3.76 ± 0.23 y) were significantly (*P* < 0.001) greater in the extracted group than in the non-extracted group. Meanwhile, the number of dental check-ups was significantly lower (*P* < 0.001) (Figure 5).

### Correlations between tooth extraction and age and dental check-up parameters

**Figure 6:**
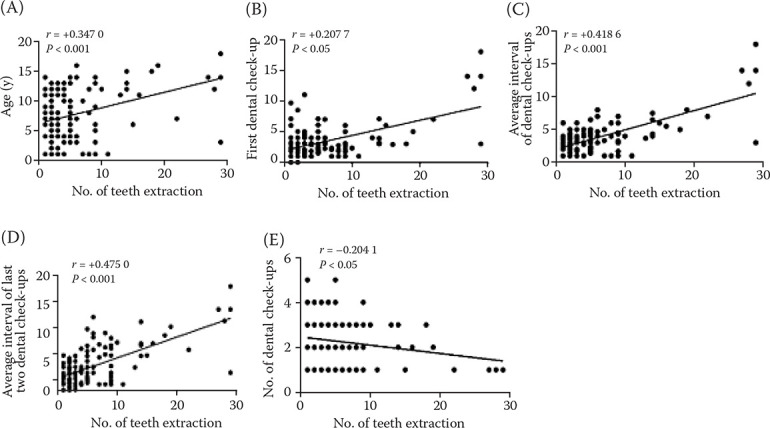
Spearman’s rank correlation between the tooth extraction and the age of the dogs (A), age at first dental check-up (B), average interval of the dental check-ups (C), average interval of the last two dental check-ups (D), and total number of the dental check-ups (E)

As shown in Figure 6, the age of the dogs (*r* = +0.347; *P* < 0.001) (A), the age at the first dental check-up (*r* = +0.207 7; *P* < 0.05) (B), the average interval of the dental check-ups (*r* = +0.418 6; *P* < 0.001) (C), and average interval of the last two dental check-ups (*r* = +0.475 0; *P* < 0.001) (D) showed a significant positive correlation with the number of teeth extracted. On the contrary, a significant negative correlation was observed between the number of teeth extracted and the number of dental check-ups (*r* = −0.204 1; *P* < 0.05) (E).

## DISCUSSION AND CONCLUSION

There are many reports in the veterinary field on periodontal disease in dogs (Kortegaard et al. 2008; Wallis et al. 2019; Wallis and Holcombe 2020), but this is the first retrospective study on dental extractions in dogs in Seoul, Republic of Korea. This study aimed to document the frequency of dental extractions in dogs in Seoul, Republic of Korea, to assess the current condition of canine dental health, and to determine preventive strategies for tooth extraction. The objective was to enhance the awareness of pet owners and doctors by identifying any correlations between the studied parameters. In the present study, teeth were extracted from 78.32% of the dogs that were examined radiographically, and the average number of teeth extracted was 5.85 ± 0.54. Multiple teeth were extracted from 86.15% of the dogs, and ten or more were extracted from 27.68% of the dogs. This can be compared with the typical one to three teeth extracted from humans (Passarelli et al. 2020). These alarming results indicate that dental health is a low priority in the veterinary field. Furthermore, we found that the age at the first dental check-up, the average interval between dental check-ups, and the average interval between the last two dental check-ups showed significant positive correlations with tooth extraction. Meanwhile, the number of dental check-ups was negatively correlated with the extraction. The current investigation suggests that regular dental examinations are the primary determinants of dental health.

The age range of the dogs undergoing tooth extraction was 1 to 18 years, with an average age of 7.76 ± 0.38 y. Interestingly, the highest proportion of teeth (36.36% of dogs) was extracted in the age group of 10 to 16 y, and the lowest proportion (9.85% of dogs) was extracted in the age group younger than 2 y. Additionally, a significant positive correlation was found between the extraction time and the age (*r* = +0.347; *P* < 0.001). Many studies have demonstrated that the prevalence and intensity of periodontal disease notably increase with age (Hoffmann and Gaengler 1996; Kortegaard et al. 2008; Marshall et al. 2014; Wallis et al. 2018; Wallis et al. 2019). This may be related to a reduced immunological function and suboptimal nutritional status associated with ageing (Marshall et al. 2014; Wallis and Holcombe 2020). Lower calcium levels in aged dogs, which potentially augment the probability of periodontal disease, may act as a predisposing factor for periodontal disease (Carreira et al. 2015). The average age at the first dental check-up was 3.48 ± 0.28 y here, younger than that in a previous study (61.8 ± 44.1 months) (Wallis et al. 2021). If a dog has no documented evidence of prior dental cleaning and polishing, the probability of periodontal disease diagnosis is higher (Wallis et al. 2021). Although there was no significant difference in age between the extraction and non-extraction groups, the age at the first dental check-up (*P* < 0.05), average interval of the dental check-ups (*P* < 0.001), and average interval of the last two dental check-ups (*P* < 0.001) were significantly greater in the extraction group than in the non-extraction group. Meanwhile, dental check-ups were significantly fewer (*P* < 0.05). Therefore, regular dental check-ups are necessary to prevent dental diseases.

Of the dogs undergoing extraction in the present study, 62.31% were male and 37.69% were female. These results are consistent with those of a previous report (Wallis et al. 2021) of periodontal disease in 52.7% male and 47.3% female dogs. Male dogs show greater aggressiveness and boldness than female dogs (Scandurra et al. 2018), which may be a predisposing factor for dental injuries and periodontitis. Additionally, 95.38% of the dogs were neutered in the tooth extraction group. A previous study also found that 70.8% of dogs were neutered (Wallis et al. 2021), which is comparatively lower than our report. This finding is supported by a previous study (Marshall et al. 2014), which reported that periodontal disease in neutered Miniature Schnauzer dogs was higher than in non-neutered dogs. In general, the higher percentage of neutered dogs in the present study might be due to the higher percentage of neutered dogs in Seoul, Republic of Korea, compared to other countries (Jeong et al. 2019; Seo et al. 2020). Neutering before the completion of bone growth can affect bone lengthening and joint development (Oberbauer et al. 2019; Seo et al. 2020). Therefore, further studies are needed to evaluate the effects of neutering on the cementum, periodontal ligament, and alveolar bone, which are the active components of dental health.

Among the 22 dog breeds in this study, the most frequently reported were Poodles (24.62%), Maltese (10.00%), and mixed breeds (9.23%). These results support a previous report indicating that Toy Poodles have notably thinner gingiva and alveolar bones than other small- and medium-sized dog breeds (Wallis and Holcombe 2020). Furthermore, a thinner gingiva is associated with more frequent periodontal diseases (Harvey 2005). The differences between studies may be due to differences in the regions or countries studied. Miniature Schnauzers are significantly likely to develop periodontitis, with 98% experiencing some abnormal periodontal conditions within 30 weeks of ceasing tooth brushing (Marshall et al. 2014). Another study showed that periodontal diseases most strongly affect Yorkshire Terriers, representing 25.2% of the cases, followed by Cocker Spaniels (12.8%) and Jack Russell Terriers (9.5%) (O’Neill et al. 2014). However, most dogs from the Republic of Korea are small breeds (Jeong et al. 2019; Seo et al. 2020). Variations in genetic factors and oral morphology have also been suggested as predisposing factors for periodontal disease (Wallis and Holcombe 2020). Smaller dogs have relatively larger teeth than larger dogs and are more prone to malocclusion and dental disorders (Wallis and Holcombe 2020; Wallis et al. 2021).

This is the first report of location-specific tooth extraction in dogs. The most commonly extracted teeth were PM2 (20.11%), followed by PM3 (19.19%), PM4 (12.09%), and PM1 (9.72%). The challenge for these teeth lies in effectively eliminating plaque using conventional brushing techniques. Oral hygiene is uncommon in dogs. Previous studies have also shown a higher frequency of periodontal disease in premolars and molars, especially those exposed to PM4 and PM1 (Kortegaard et al. 2008; Wallis and Holcombe 2020). Some studies have indicated that premolars experience the highest occurrence of periodontal diseases (Hoffmann and Gaengler 1996; Wallis et al. 2019). Meanwhile, others have suggested that incisors are particularly susceptible to dental diseases (Marshall et al. 2014; Wallis et al. 2018). Although extracted teeth in the mandible (*n* = 388) are higher than in the maxilla (*n* = 373) in this study, there was no significant difference in the number of extracted teeth between the maxilla and mandible. This does not disprove a previous report that periodontal diseases are more common in the mandible than in the maxilla (Marshall et al. 2014). However, multiple studies have indicated a higher prevalence of the disease in maxillary teeth than in mandibular teeth (Kortegaard et al. 2008; Wallis et al. 2019; Wallis and Holcombe 2020). These disparities are attributable to variations in the population and the methodology. The extracted PM2 and PM3 on the left side (maxilla 43 and 41, respectively; mandible 46 and 43, respectively) were markedly higher than those on the right side (maxilla 30 and 31, respectively; mandible 34 and 31, respectively) in this study. This is the first difference observed in this study and is difficult to explain due to the scarcity of previous reports. Additional studies are required to address these limitations in future research.

Taken together, aged dogs are more susceptible to dental disorders, and male dogs have a higher prevalence of dental disorders than female dogs. The premolar and molar teeth were the most affected, especially PM2, PM3, and PM4. Most dogs in Seoul, Republic of Korea, have undergone the extraction of multiple teeth. The present findings indicate that regular dental check-ups are the key factor in dental health. Veterinarians and owners can use this knowledge to enhance the efficacy of therapies and prevent tooth extractions.
